# Efficacy and safety of 3D print-assisted surgery for the treatment of pilon fractures: a meta-analysis of randomized controlled trials

**DOI:** 10.1186/s13018-018-0976-x

**Published:** 2018-11-12

**Authors:** Jianzhong Bai, Yongxiang Wang, Pei Zhang, Meiying Liu, Peian Wang, Jingcheng Wang, Yuan Liang

**Affiliations:** 1 Dalian Medical University, Dalian, 116044 Liaoning China; 2 Clinical Medical College, Yangzhou University, Yangzhou, 225001 China; 3 Heze Mudan People’s Hospital, Heze, 274000 China

**Keywords:** Three-dimensional, 3D printing, Computer-assisted, Pilon fractures, Surgery

## Abstract

**Objective:**

To compare the effects of 3D print-assisted surgery and conventional surgery in the treatment of pilon fractures.

**Methods:**

PubMed, Embase, Web of Science, CNKI, CBM, and WanFang data were searched until July 2018. Two reviewers selected relevant studies, assessed the quality of studies, and extracted data. For continuous data, a weighted mean difference (WMD) and 95% confidence intervals (CI) were used. For dichotomous data, a relative risk (RR) and 95% CI were calculated as the summary statistics.

**Results:**

There were seven randomized controlled trials (RCT) enrolling a total of 486 patients, 242 patients underwent 3D print-assisted surgery and 244 patients underwent conventional surgery. The pooled outcomes demonstrate 3D print-assisted surgery was superior to conventional surgery in terms of operation time [WMD = − 26.16, 95% CI (− 33.19, − 19.14), *P* < 0.001], blood loss [WMD = − 63.91, 95% CI (− 79.55, − 48.27), *P* < 0.001], postoperative functional scores [WMD = 8.16, 95% CI (5.04, 11.29), *P* < 0.001], postoperative visual analogue score (VAS) [WMD = − 0.59, 95% CI (− 1.18, − 0.01), *P* = 0.05], rate of excellent and good outcome [RR = 1.20, 95% CI (1.07, 1.34), *P* = 0.002], and rate of anatomic reduction [RR = 1.35, 95% CI (1.19, 1.53), *P* < 0.001]. However, there was no significant difference between the groups regarding the rate of infection [RR = 0.51, 95% CI (0.20, 1.31), *P* = 0.16], fracture union time [WMD = − 0.85, 95% CI (− 1.79, 0.08), *P* = 0.07], traumatic arthritis [RR = 0.34, 95% CI (0.06, 2.09), *P* = 0.24], and malunion [RR = 0.34, 95% CI (0.06, 2.05), *P* = 0.24].

**Conclusions:**

Our meta-analysis demonstrates 3D print-assisted surgery was significantly better than conventional surgery in terms of operation time, blood loss, postoperative functional score, postoperative VAS, rate of excellent and good outcome, and rate of anatomic reduction. Concerning postoperative complications, there were no significant differences between the groups.

## Introduction

Pilon fractures are usually caused by high energy trauma, accompanied by multiple metaphyseal fragments, displaced intra-articular comminution, and severe soft tissue injuries: this is a substantial problem for experienced orthopedic surgeons [1, 2]. The purpose of surgical treatment is an anatomic reduction of the articular fragments, firm fixation, and early functional exercise [3, 4]. However, postoperative complications seriously affect the effects of surgery, such as severe pain, skin necrosis, malunion, implant failure, joint stiffness, and even posttraumatic arthritis [5, 6]. Therefore, it is necessary to seek a new method to reduce postoperative complications and improve the outcomes of surgery.

Recently, 3D printing technology has developed rapidly in the medical field [7], primarily using a 3D digital model to build a 1:1 fracture model based on the patient’s imaging data. Furthermore, surgeons can perform a pre-operation to identify unforeseen problems during surgery that could assist in formulation of preoperative planning, simulation of the surgical procedure, and achievement of better surgical outcomes [8]. However, there are no relevant meta-analyses or clinical guides to assess the effects of 3D print-assisted surgery for the treatment of pilon fractures. It is unclear whether 3D print-assisted surgery can significantly improve the postoperative outcomes of patients compared to conventional surgery. Therefore, we performed this meta-analysis to identify the issue and then provided a better treatment strategy for clinicians.

## Methods

We carried out this meta-analysis strictly according to the Preferred Reporting Items for Systematic Reviews and Meta-Analysis (PRISMA) statement [9] and the Cochrane Collaboration guidelines.

### Search strategy

PubMed, Embase, Web of Science, CNKI, CBM, and WanFang data were searched until July 2018. Besides, we manually searched the reference lists of all included relevant publications to identify potential studies. We considered articles published in any language. The following keywords were adopted in the database search: “pilon fractures,” “3D printing,” “computer-assisted,” and “surgery.” The Boolean operators were used to combine them.

### Study selection and eligibility criteria

The inclusion criteria were as follows: (1) pilon fractures diagnosed by validated screening or diagnostic instruments, (2) the study compared 3D print-assisted surgery with conventional surgery for the treatment of pilon fractures, (3) the study design was randomized controlled trial (RCT), (4) Chinese articles included must have title and abstract in English, and (5) the study contained at least one of the following indicators: operation time, blood loss, postoperative functional score, rate of excellent and good outcome, rate of anatomic reduction, or postoperative complications. The exclusion criteria were as follows: (1) other types of fractures, (2) studies provided insufficient data, and (3) case report, review, commentary, or study only included an abstract (Table 1).

**Table 1:** Characteristics of included studies

Studies	Year	Study year	Groups	Sample size	Age ± mean (year)	Pilon fracture classification
Huang et al.	2015	2008–2013	3D	31	48.6	RA: I 9, II 12, III 10
			C	30	48.6	RA: I 7, II 15, III 8
Tang et al.	2015	2012–2014	3D	32	38.4 ± 2.8	RA: II 12, III 20
			C	32	37.2 ± 2.4	RA: II 15, III 17
Fan et al.	2016	2014–2015	3D	50	43.5 ± 3.5	RA: II 20, III 30
			C	50	43.5 ± 3.5	RA: II 21, III 29
Li et al.	2016	2013–2014	3D	30	34.8 ± 6.0	AO:13 C2, 17 C3
			C	30	35.8 ± 6.2	AO:12 C2, 18 C3
Gu et al.	2017	2011–2015	3D	36	38.9 ± 5.9	RA: II 15, III 21
			C	36	39.6 ± 5.5	RA: II 12, III 24
Ou et al.	2017	NR	3D	18	37.4 ± 3.7	RA: II 10, III 8
			C	18	38.4 ± 3.5	RA: II 9, III 9
Zheng et al.	2018	2013–2016	3D	45	41.2 ± 9.3	AO:5 C1, 14 C2, 26 C3
			C	48	42.5 ± 9.0	AO: 8 C1, 17 C2, 23 C3

*3D* 3D print-assisted surgery, *C* conventional surgery, *RA* Ruedi-Allgower, *NR* no report

### Data extraction

Two reviewers performed data extraction. The following information was extracted from eligible studies: author, year, study design, sample size, age, postoperative outcomes, and classification of pilon fractures. Any disagreements were resolved by discussion to reach a consensus. All extracted data were entered into a predefined standardized Excel (Microsoft Corporation, USA) file carefully.

### Quality assessment

We evaluated the quality of the RCTs according to the methods of the 12-item scale [10]. Each item was scored “Yes,” “Unclear,” or “No.” A study with a score of more than 7 “Yes” response was considered as of high quality, 5–7 was considered as of moderate quality, and 0–4 was considered as of low quality.

### Statistical analysis

Statistical analyses were performed by using Revman 5.3 software. For continuous outcomes, weighted mean difference (WMD) with 95% CI was used. For dichotomous data, relative risk (RR) with 95% CI was calculated as the summary statistics. *P* ≤ 0.05 was regarded as statistically significant. The *I*^2^ statistic assessed statistical heterogeneity, with *I*^2^ value more than 50% indicating significant heterogeneity, the random-effects model was used to do the analysis; otherwise, the fixed-effects model was used. In addition, sensitivity analyses were conducted to insure the accuracy of the outcomes.

## Results

### Search result

**Fig. 1:**
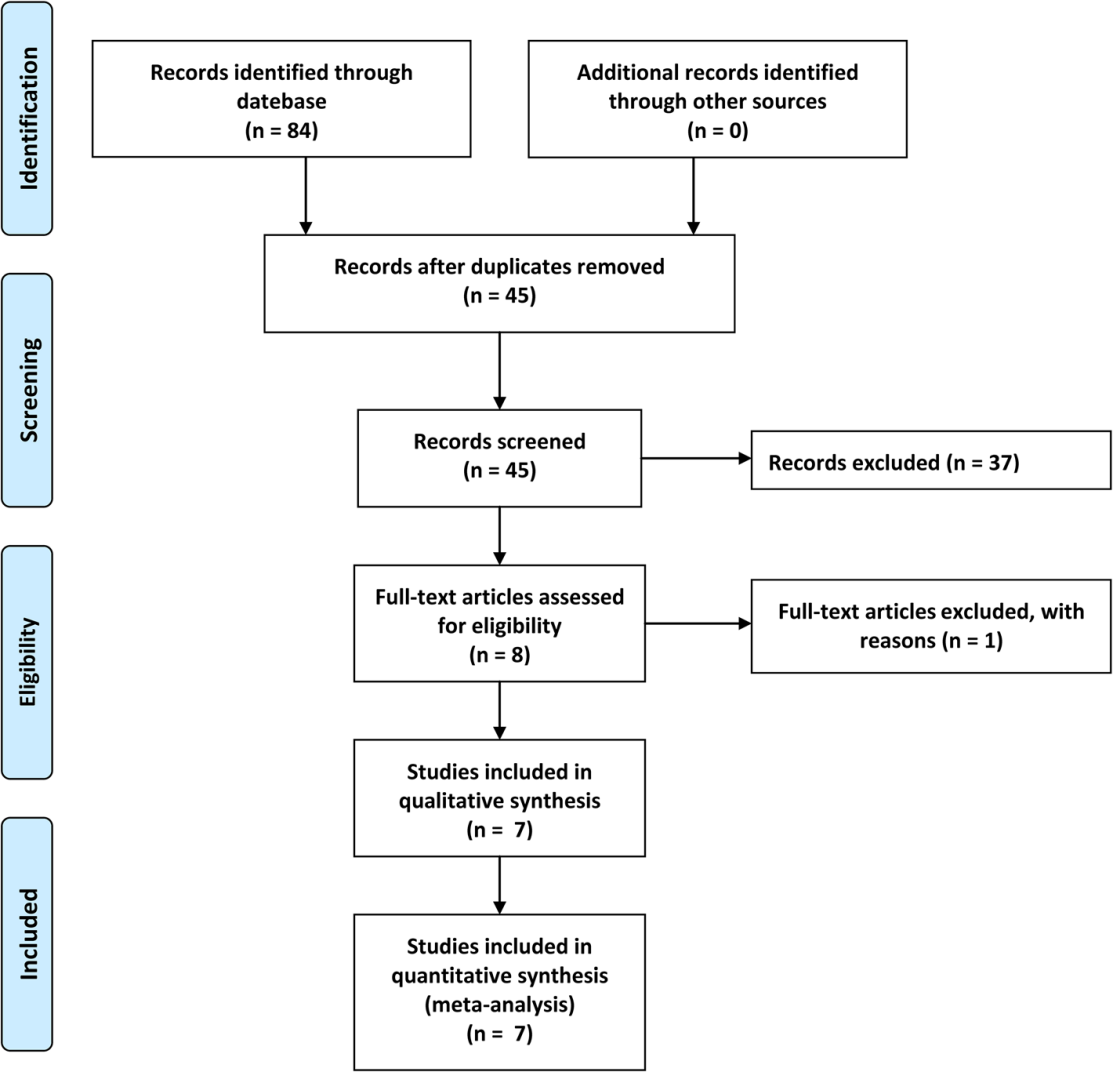
The flow chart of studies selecting

A total of 84 potentially relevant references were found. We removed 39 duplicate studies. By scanning the titles and abstracts, 37 studies were excluded from the analysis. After full texts were carefully read according to eligibility, one study was excluded because it was not an RCT [11]. Finally, seven studies were included in quantitative synthesis [12–18]. The characteristics of all included studies are shown in Table 1. Details of the study selection process are shown in Fig. 1.

### Quality assessment

The details of the quality assessment of included studies are shown in Table 2. Five studies [12, 13, 15, 17, 18] were of high quality, and two studies [14, 16] were of moderate quality. The randomization methods were explicitly introduced in five studies [12, 13, 15, 17, 18]. No study reported blinding of outcome assessment. However, all of the included studies were reported with complete outcome data.

**Table 2:** The 12-item appraisal scores for the RCTs

Studies	Randomized adequately^a^	Allocation concealed	Patient blinded	Care provider blinded	Outcome assessor blinded	Acceptable drop-out rate^b^	ITT analysis^c^	Avoided selective reporting	Similar baseline	Similar or avoided cofactor	Patient compliance	Similar timing	Quality^d^
Huang et al	Yes	Yes	Unclear	Unclear	Unclear	Yes	Yes	Yes	Yes	Unclear	Yes	Yes	High
Tang et al	Yes	Yes	Unclear	Unclear	Unclear	Yes	Yes	Yes	Yes	Unclear	Yes	Yes	High
Fan et al	Yes	Yes	Unclear	Unclear	Unclear	Yes	Yes	Yes	Yes	Unclear	Yes	Yes	High
Li et al	No	Unclear	Unclear	Unclear	Unclear	Yes	Yes	Yes	Yes	Unclear	Yes	Yes	Moderate
Gu et al	Yes	Yes	Unclear	Unclear	Unclear	Yes	Yes	Yes	Yes	Unclear	Yes	Yes	High
Ou et al	Unclear	Yes	Unclear	Unclear	Unclear	Yes	Yes	Yes	Yes	Unclear	Yes	Yes	Moderate
Zheng et al	Yes	Yes	Unclear	Unclear	Unclear	Yes	Yes	Yes	Yes	Unclear	Yes	Yes	High

^a^Only if the method of sequence made was explicitly introduced could get a “Yes”

^b^Drop-out rate < 20% could get a “Yes,” otherwise “No”

^c^*ITT* intention-to-treat, only if all randomized participants were analyzed in the group, they were allocated to could receive a “Yes”

^d^“Yes” items more than 7 means “High”; more than 4 but no more than 7 means “Moderate”; no more than 4 means “Low”

### Clinical outcomes

#### Operation time (mins)

**Fig. 2:**
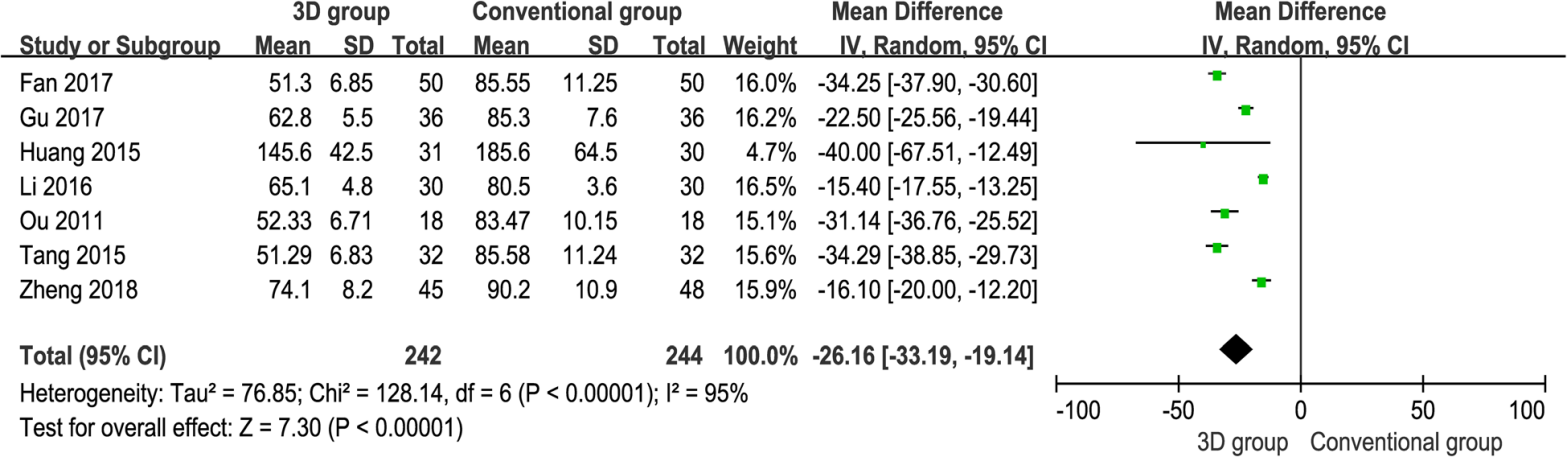
The forest plot for operation time

The operation time was reported in seven studies [12–18], and the pooled results demonstrated that the 3D print-assisted surgery group had a significantly shorter operation time than did the conventional surgery group [WMD = − 26.16, 95% CI (− 33.19, − 19.14), *P* < 0.001, *I*^2^ = 95%, Fig. 2].

#### Blood loss (ml)

**Fig. 3:**
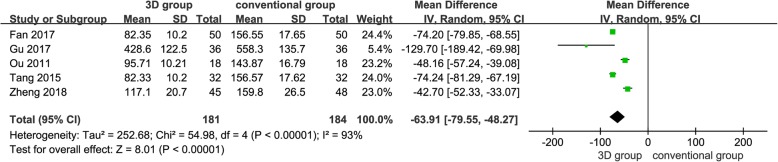
The forest plot for blood loss

Five studies [12, 13, 15–17] provided available data, and the pooled results demonstrated that the 3D print-assisted surgery group had a significantly less blood loss than the conventional surgery group [WMD = − 63.91, 95% CI (− 79.55, − 48.27), *P* < 0.001, *I*^2^ = 93%, Fig. 3].

### Postoperative functional scores

**Fig. 4:**
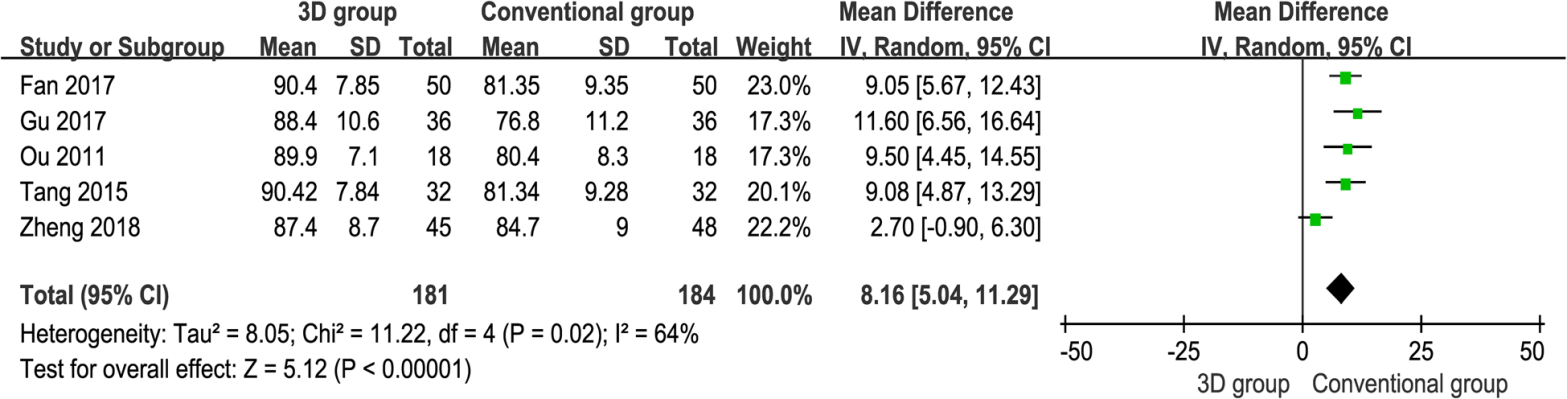
The forest plot for postoperative functional score

Five studies [12, 13, 15–17] provided available data, and the pooled results demonstrated that the 3D print-assisted surgery group had a significantly higher functional score than did the conventional surgery group [WMD = 8.16, 95% CI (5.04, 11.29), *P* < 0.001, *I*^2^ = 64%, Fig. 4].

### The rate of excellent and good outcomes

**Fig. 5:**
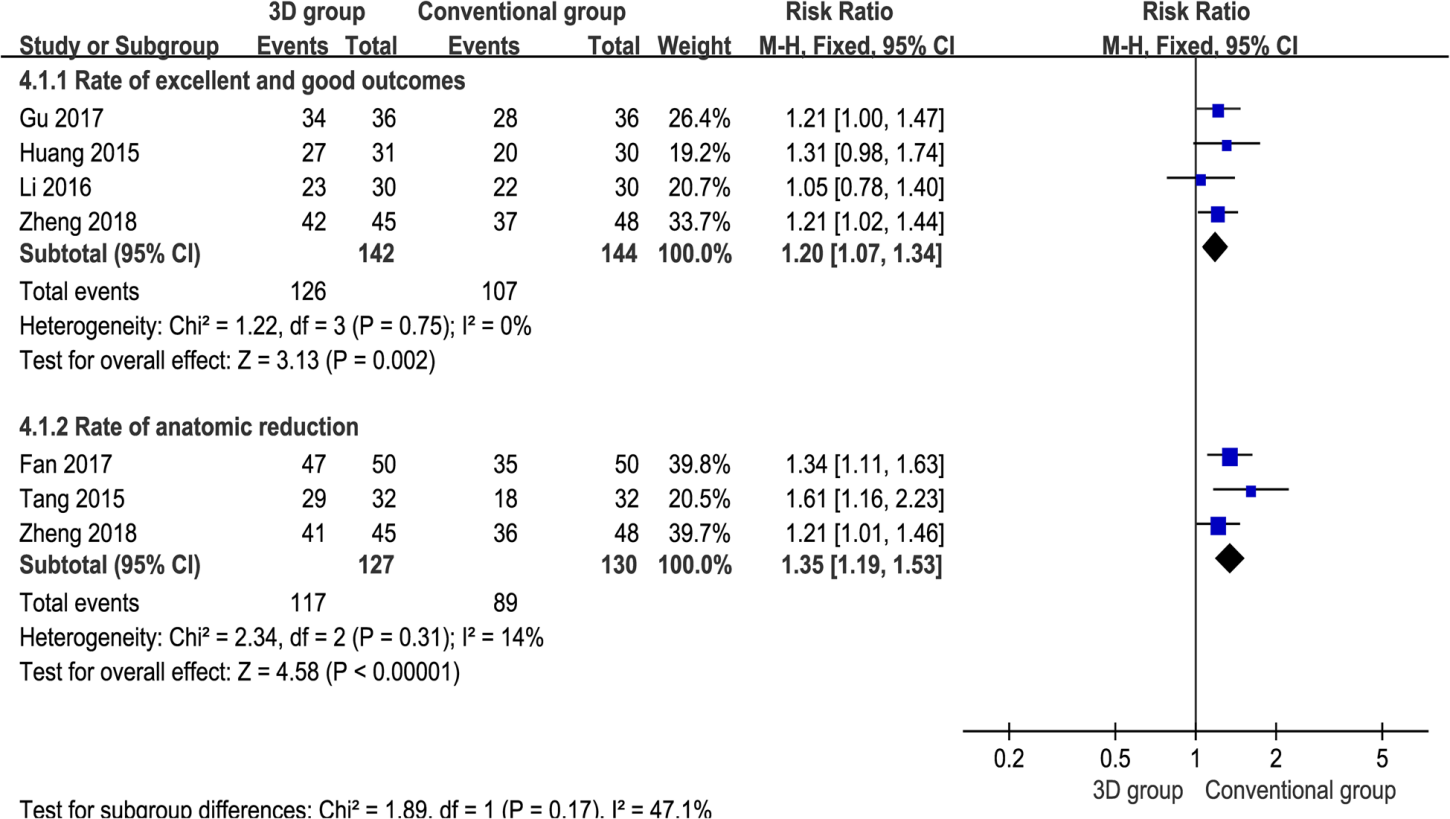
The forest plot for rate of excellent and good outcome and rate of anatomic reduction

Four studies [14, 15, 17, 18] provided available data, and the pooled results demonstrated that the 3D print-assisted surgery group had a higher rate of excellent and good outcomes than did the conventional surgery group [RR = 1.20, 95% CI (1.07, 1.34), *P* = 0.002, *I*^2^ = 0%, Fig. 5].

### The rate of anatomic reduction

Three studies [12, 13, 17] provided available data, and the pooled results demonstrated that the 3D print-assisted surgery group had a higher rate of anatomic reduction than the conventional surgery group [RR = 1.35, 95% CI (1.19, 1.53), *P* < 0.001, *I*^2^ = 14%, Fig. 5].

### Fracture union time (month)

**Fig. 6:**
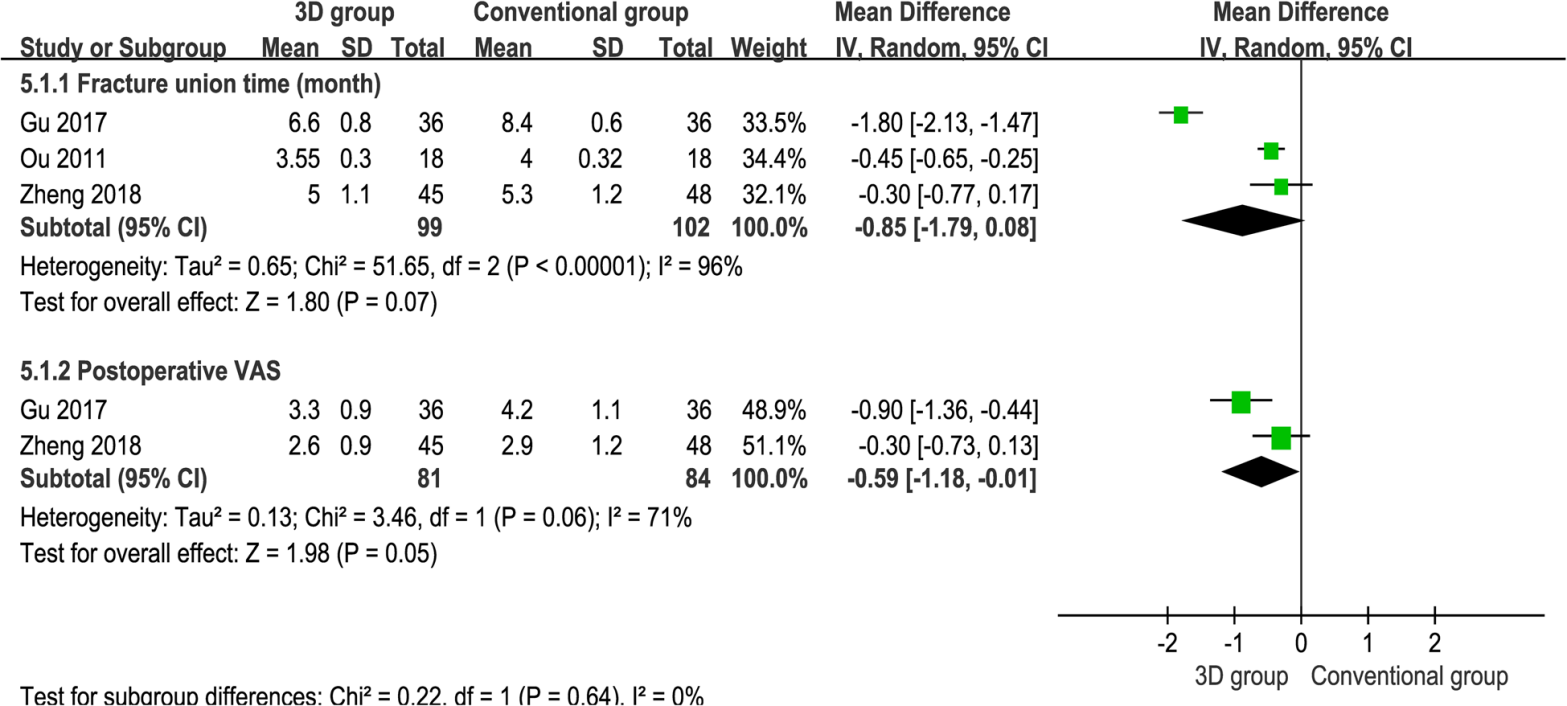
The forest plot for fracture union time and postoperative VAS

Three studies [15–17] provided available data concerning fracture union time, and the pooled outcomes demonstrated that there was no significant difference between the groups [WMD = − 0.85, 95% CI (− 1.79, 0.08), *P* = 0.07, *I*^2^ = 96%, Fig. 6].

### Postoperative VAS

Two studies [15, 17] provided available data, and the pooled outcomes demonstrated that 3D print-assisted surgery group had a lower VAS than the conventional surgery group [WMD = − 0.59, 95% CI (− 1.18, − 0.01), *P* = 0.05, *I*^2^ = 71%, Fig. 6].

### Traumatic arthritis

**Fig. 7:**
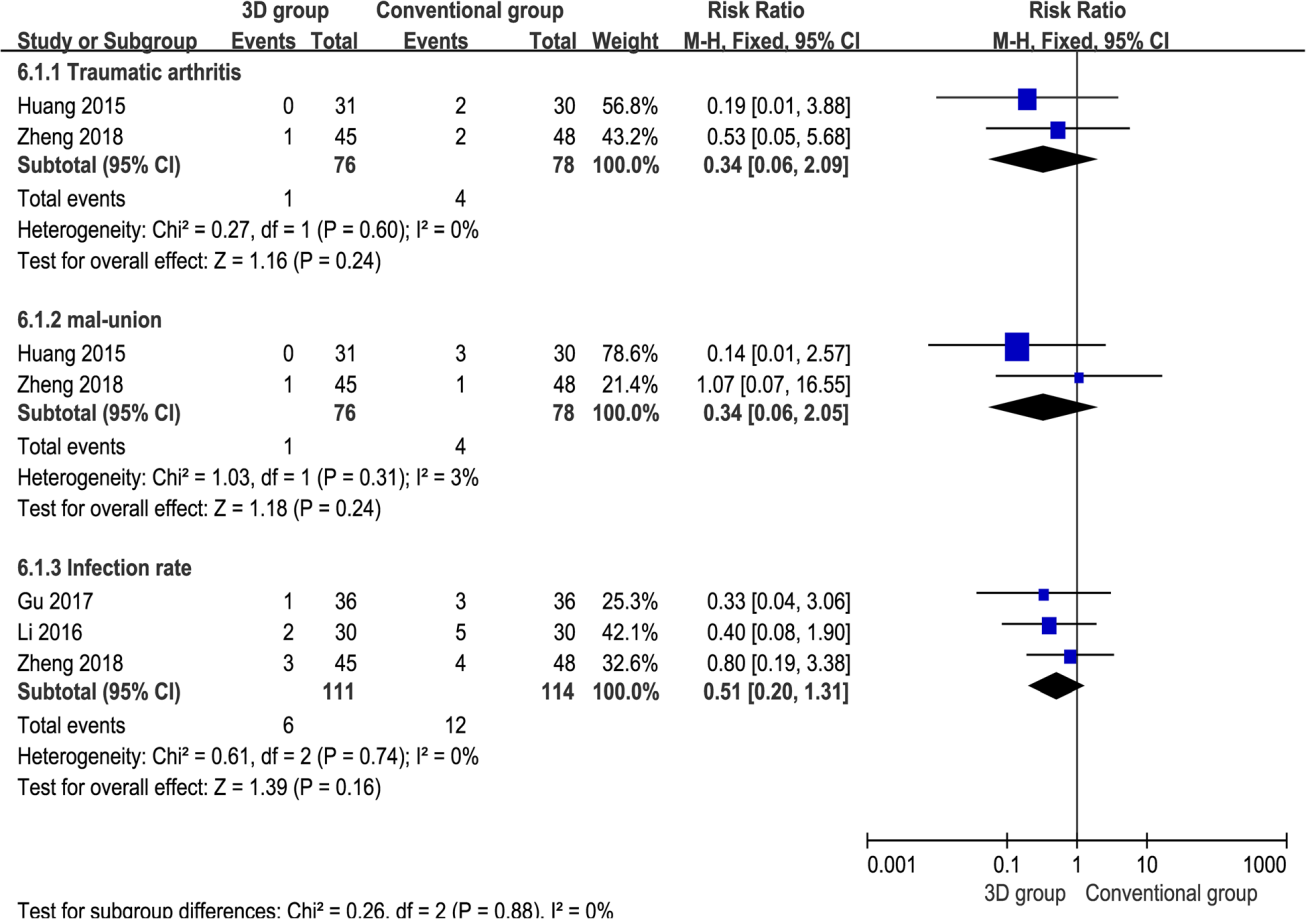
The forest plot for rate of traumatic arthritis, malunion, and infection rate

Two studies [17, 18] provided available data, and the pooled results demonstrated that two surgical methods have a similar effect regarding the rate of traumatic arthritis [RR = 0.34, 95% CI (0.06, 2.09), *P* = 0.24, *I*^2^ = 0%, Fig. 7].

### Malunion

Two studies [17, 18] provided available data regarding malunion, and the pooled results demonstrated there was no significant difference between the groups [RR = 0.34, 95% CI (0.06, 2.05), *P* = 0.24, *I*^2^ = 3%, Fig. 7].

### Infection rate

Three studies [14, 15, 17] provided available data concerning infection rate, and the pooled results demonstrated that the 3D print-assisted surgery group had a lower infection rate than the conventional surgery group, but there is no significant difference between the groups [RR = 0.51, 95% CI (0.20, 1.31), *P* = 0.16, *I*^2^ = 0%, Fig. 7].

### Sensitivity analysis

Due to fewer studies were included in some outcomes, we only performed sensitivity analysis on the results of operation time, blood loss, and postoperative functional score. These outcomes all remained stable after the exclusion of each study once a time.

## Discussion

### Main findings

Our meta-analysis demonstrated that the 3D print-assisted surgery was significantly better than the conventional surgery concerning operation time, blood loss, postoperative functional score, postoperative VAS, rate of excellent and good outcome, and rate of anatomic reduction. Although the 3D print-assisted surgery group had a lower incidence rate than the conventional surgery group concerning infection rate, traumatic arthritis, and malunion, there were no significant differences between the groups.

It is approximated that pilon fractures constitute 1% of all lower extremity fractures and 5–10% of tibia fractures [19]. Most pilon fractures require surgery, and the main purpose is to firmly fix the intra-articular fragments and restore the length and alignment, allowing for earlier weight bearing and functional exercise [20]. Orthopedists usually formulate surgical plans based on X-ray, CT, or other examination outcomes with conventional surgery [21]. However, pilon fractures are severely comminuted fractures, and the ankle joints are often accompanied by severe collapse and loss of bone. Conventional imaging outcomes cannot directly display the specific shape of the fracture, and even sometimes omit occult fractures. The surgeon continues surgery based on clinical experience when the intraoperative condition is not consistent with the expected situation during surgery, which possibly leads to change of the surgical plan, prolong the operation time, increase the blood loss, aggravate the soft tissue injury, and even cause the failure of the operation. Therefore, it is critical for surgeons to perform a pre-surgery based on 3D printing model. They can predict the problems that may be encountered during the operation, such as the optimal surgical approach, matched implant. Therefore, this surgical method shortens the operation time and improves the effects of surgery [22]. In addition, the surgeon can adequately communicate with patients using this vivid fracture model [23].

Although 3D printing technology promotes the development of orthopedic surgery, it has some certain limitations, such as increasing the economic burden of patients. Besides, 3D printing technology requires high requirements, complicated operating technique, and expensive 3D printing instruments that limit the promotion of this technology. Furthermore, for some complex intra-articular fractures, reconstruction and printing of 3D models increase preoperative preparation time, so this technique is not suitable for emergency surgery. Another disadvantage of 3D printing technology is that it cannot be displayed for soft tissues, such as vasculars and nerves.

Currently, there remains a lack of attention to the treatment of pilon fractures with 3D print-assisted surgery, and to the best of our knowledge, there has been no meta-analysis of the comparison between the methods. Our meta-analysis demonstrated that 3D print-assisted surgery has advantages in terms of operation time, blood loss, and functional scores, similar to the previous studies [12–17]. However, both groups had similar infection rates, the pooled results consistent with the results of Li et al. and Zheng et al. [14, 17]. In theory, 3D print-assisted surgery has shorter operative time and less blood loss, so the infection rate should be lower, which requires a large sample of RCTs to update this conclusion.

### Limitations

Although this was the first meta-analysis to compare 3D print-assisted surgery with conventional surgery for the treatment of pilon fractures based on seven RCTs, there was a small sample size of included studies, possibly affecting the accuracy of our conclusions. Besides, this meta-analysis had a higher heterogeneity in some pooled outcomes; unequal levels of regional medical care, varying follow-up time, different levels of the operators, and degree of patient injury may contribute it.

## Conclusions

Our meta-analysis demonstrates 3D print-assisted surgery was significantly better than the conventional surgery in terms of operation time, blood loss, postoperative functional score, postoperative VAS, rate of excellent and good outcome, and rate of anatomic reduction. Although the 3D print-assisted surgery group had a lower incidence rate than the conventional surgery group concerning infection rate, traumatic arthritis, and malunion, there were no significant differences between the groups. Future large-volume, well-designed RCTs with extensive follow-up are awaited to confirm and update the findings of this analysis.

## Abbreviations

3D: Three-dimensional
CI: Confidence interval
RCT: Randomized controlled trial
RR: Relative risk
VAS: Visual analogue score
WMD: Weighted mean difference
